# Inconsistency between non-invasive prenatal testing (NIPT) and conventional prenatal diagnosis due to confined placental and fetal mosaicism: Two case reports

**DOI:** 10.3389/fmed.2022.1063480

**Published:** 2022-12-15

**Authors:** Kyung Min Kang, Soo Hyun Kim, Ji Eun Park, Hyunjin Kim, Hee Yeon Jang, Minyeon Go, So Hyun Yang, Sang Woo Ryu, Sung Mi Bae, Dong Hyun Cha, Sung Han Shim

**Affiliations:** ^1^Center for Genome Diagnostics, CHA Biotech Inc., Seoul, Republic of Korea; ^2^Department of Obstetrics and Gynecology, CHA Gangnam Medical Center, CHA University, Seoul, Republic of Korea; ^3^Department of Biomedical Science, College of Life Science, CHA University, Seongnam, Republic of Korea; ^4^Potato & Snowman Infertility Women’s Clinic, Seoul, Republic of Korea

**Keywords:** placenta-derived cell free DNA, whole genome sequencing, non-invasive prenatal testing, confined placental mosaicism, mass parallel sequencing

## Abstract

We aimed to identify the causes of inconsistent results between non-invasive prenatal testing (NIPT) and invasive testing methods for trisomy 21. In the first case, NIPT was performed at 11 weeks of pregnancy, and the result showed a high risk of trisomy 21 [fetal fraction (FF) = 6.98%, 21 chromosome Z-score = 3.6]. The patient underwent quantitative fluorescent (QF)-PCR and karyotyping at 14 + 0 weeks of pregnancy through CVS showing mosaicism of 47, XX, + 21[11] and 46, XX [39] in karyotyping. The patient underwent amniocentesis at 15 + 6 weeks, showing a normal pattern in QF-PCR and 46, XX karyotyping in long term culture. The second case underwent NIPT at 16 + 5 weeks of pregnancy (FF = 7.52%, 21 chromosome Z-score = 2.503). She underwent an invasive test at 19 weeks through amniotic fluid sampling. As a result, trisomy 21 was detected by QF-PCR, and mosaicism of XX, +21[22]/46, XX [4] was identified by karyotyping. Despite significant advances in fetal chromosome analysis using NIPT, invasive testing is still needed as placenta-derived DNA does not reflect 100% fetal genetic information. Placental mosaicism can be detected by NIPT, but more research is needed to increase its sensitivity. Therefore, if the NIPT result is positive, an invasive test can confirm the result, and continuous monitoring is required even if the NIPT result is negative.

## 1 Introduction

Prenatal screening and diagnosis of fetal chromosomal aneuploidy have become common among pregnant women (1–3). These screening methods, such as fetal ultrasound and maternal serum biomarker screening, have detection rates of 60–90% and a false positive rate of 5% (1, 4). If these tests show a high risk of fetal chromosomal aneuploidy, pregnant women are recommended to undergo invasive diagnostic tests such as chorionic villus sampling (CVS) at 12–13 weeks of gestation and amniocentesis at 15–16 weeks of gestation (1, 2). Although these tests are valuable diagnostic tools because of their high accuracy, they are associated with a risk of miscarriage between 0.5 and 1.0% (5, 6). Since the discovery of placenta-derived cell-free DNA (cfDNA) in the peripheral blood of pregnant women in the late 1980s, various attempts have been made to use it for prenatal genetic screening (7–10).

Therefore, detection of fetal chromosomal aneuploidy using cfDNA is expected to be an alternative to invasive tests. Many clinical studies have successfully applied mass parallel sequencing (MPS) of maternal cfDNA using whole genome sequencing or target sequencing methods. (11, 12). A meta-analysis of the clinical validation and implementation of the non-invasive prenatal testing (NIPT) method revealed a high sensitivity and specificity (92–99%) for trisomy 21, 18, and 13 (4, 13, 14). NIPT has been recently recommended by several professional societies, such as the International Society for Ultrasound in Obstetrics and Gynecology (ISUOG) and the American College of Obstetricians and Gynecologists (ACOG), International Society of Prenatal Diagnosis (ISPD), and the Royal College of Obstetricians and Gynecologists (RCOG).

Although the genetic information of placental tissues is known to representative of the fetus in most cases, mosaicism is observed (mostly confined to the placenta) in 1–2% of karyotypes (15). Therefore, the proportion of inconsistent results due to confined placental mosaicism (CPM) observed between CVS and amniocentesis is similar to that of NIPT and amniocentesis.

Non-invasive prenatal testing is a next-generation technology with great potential as a screening tool for pregnant women. However, it is important to emphasize that NIPT is only a screening tool, not a diagnostic of fetal aneuploidy. Therefore, if the NIPT result is positive, an invasive test is required to confirm the result, and continuous observation is required even if the NIPT result is negative.

In this study, we report two cases of discrepancy between NIPT and invasive diagnostic methods and their follow-up studies for more accurate prenatal genetic counseling on NIPT results. The z-scores of two patients were showed values that did not belong to the low-risk group and the high-risk group.

## 2 Materials and methods

### 2.1 Participants

Patient 1: The 30-year old patient had a history of four pregnancies including this pregnancy and three spontaneous abortions; 47, XY, +21 in the first pregnancy, 45, X in the second pregnancy, and conjoined twin in the third pregnancy. She was naturally pregnant and nuchal translucency (NT) was 1.0 mm on ultrasonography at 15 + 6 weeks of gestation. No ultrasound abnormalities were identified.

Patient 2: The 37-year old patient had a history of five pregnancies, including this pregnancy and four spontaneous abortions; She was pregnant through *in vitro* fertilization (IVF) and nuchal translucency (NT) was 1.2 mm on ultrasonography at 16 + 5 weeks of gestation. No ultrasound abnormalities were identified.

We obtained written informed consent for participation in the study from 2 patients, and the study was approved by the institutional review board of the CHA Gangnam Medical Center, CHA University, Seoul, Korea (Approval number: GCI-2022-04-015). The studies for 1,653 data were approved by the institutional review board of the CHA Gangnam Medical Center, CHA University, Seoul, Korea (approval number: GCI-20-11).

### 2.2 Sample preparation and sequencing

Approximately 10 ml of maternal peripheral blood samples were collected in Cell-Free DNA BCT™ tubes (Streck, Omaha, NE, USA) and stored at room temperature until further processing. After centrifuging the whole blood samples at 1,200 × *g* for 10 min at 4°C, plasma was separated from the maternal cells and transferred to microcentrifuge tubes. Samples were centrifuged at 16,000 × *g* for 10 min and the supernatant was separated from residual cells, transferred to new tubes and stored at –20°C until required for analysis. For each sample, plasma cfDNA was extracted from 1 mL of plasma using the QIAamp Circulating Nucleic Acid Kit (Qiagen, Hilden, Germany). The cfDNA was used to for library preparation using the Ion Plus Fragment Library kit (Thermo Fisher, Waltham, CA, USA) according to the manufacturer’s instructions. DNA libraries were analyzed using the Ion S5™ XL System (Life Technologies, Singapore) with an average 0.3× sequencing coverage depth. A total of 12 cfDNA samples were loaded onto an Ion 540™ Chip Kit (version 2.0; Life Technologies, CA, USA). The raw reads of each sample were above 5 million, and the rates of uniquely mapped reads was above 65.0%.

### 2.3 Data and statistical analyses

Raw reads obtained from the Ion Torrent Suite software (version 5.16.1) were trimmed and filtered using Picard with default parameters. The sequence fragments were aligned and mapped to the human reference genome sequence (hg19) using the Burrows-Wheeler Aligner (BWA). The effect of GC bias was reduced and normalized using LOESS regression. The z-score for each chromosome in each sample was calculated using the mean mapped reads and the standard deviation (SD). Standard formulas for binomial distributions were used to calculate the positive predictive value (PPV) and negative predictive value (NPV). Data were analyzed using Wilson’s interval method and MedCalc version 12.1.4 (MedCalc Software Ltd., Ostend, Belgium). Samples with a fetal fraction (FF) less than 4.0% were described as no-calls and re-sampled or rejected according to the FF value. The aneuploidy of chromosome 21 was assessed according to the z-score value and identified with one of the following groups: ≥3.5 = high risk, ≥ 2.5 = intermediate risk, and between −2.5 and 2.5 = low risk.

### 2.4 DNA extraction

Genomic DNA was extracted from the amniocytes, placental tissue, and parental blood samples; 1.5 ml of amniotic fluid using InstaGene™ Matrix (Bio-Rad Laboratories, Inc., CA, USA), 200 μl of peripheral blood using QuickGene DNA blood kit (Kurabo, Osaka, Japan), and 1 mg of placental tissue using QuickGene DNA tissue kit (Kurabo) according to the manufacturer’s instructions.

### 2.5 QF-PCR and UPD test

DNA (10 ng) was amplified using Elucigene QST*R Plus v2 or QST*R-21 (Delta Diagnostics, Manchester, UK) according to the manufacturer’s instructions. The PCR products were analyzed using ABI 3500 (Applied Biosystems, CA, USA) and GeneMapper software (Applied Biosystems). Short tandem repeat (STR) markers, such as the 7 informative markers D21S11 (21q21.1), D21S1437 (21q21.1), D21S1409 (21q21.1), D21S1442 (21q21.3), D21S1435 (21q21.3), D21S1411 (21q22.3), and D21S1446 (21q22.3), were used to perform polymorphic marker analysis on chromosome 21 region to exclude uniparental disomy (UPD).

### 2.6 Cytogenetic analysis

Amniocytes were grown in Chang Medium^®^ *In Situ* (Irvine Scientific, Santa Ana, CA, USA) using the *in situ* coverslip culture method. GTG-banded metaphase chromosomes were obtained from 15 colonies and analyzed using CytoVision version 3.6 (Applied Imaging, Thunderland, UK). The results were interpreted according to the International System for Human Cytogenetic Nomenclature, 2020.

## 3 Results

### 3.1 Case 1

**FIGURE 1:**
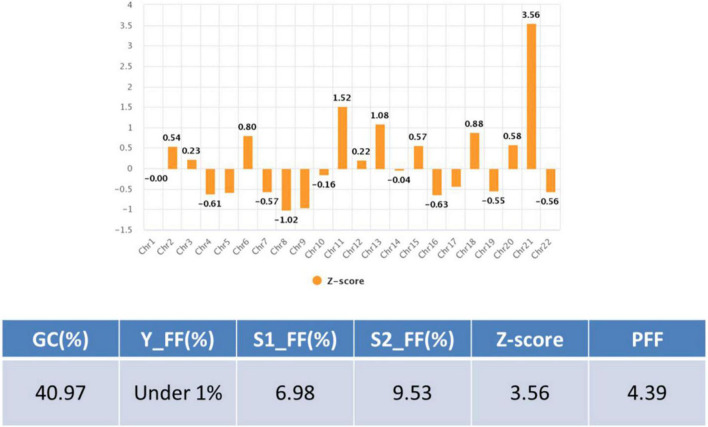
Non- invasive prenatal testing (NIPT) data in case 1.

**FIGURE 2:**
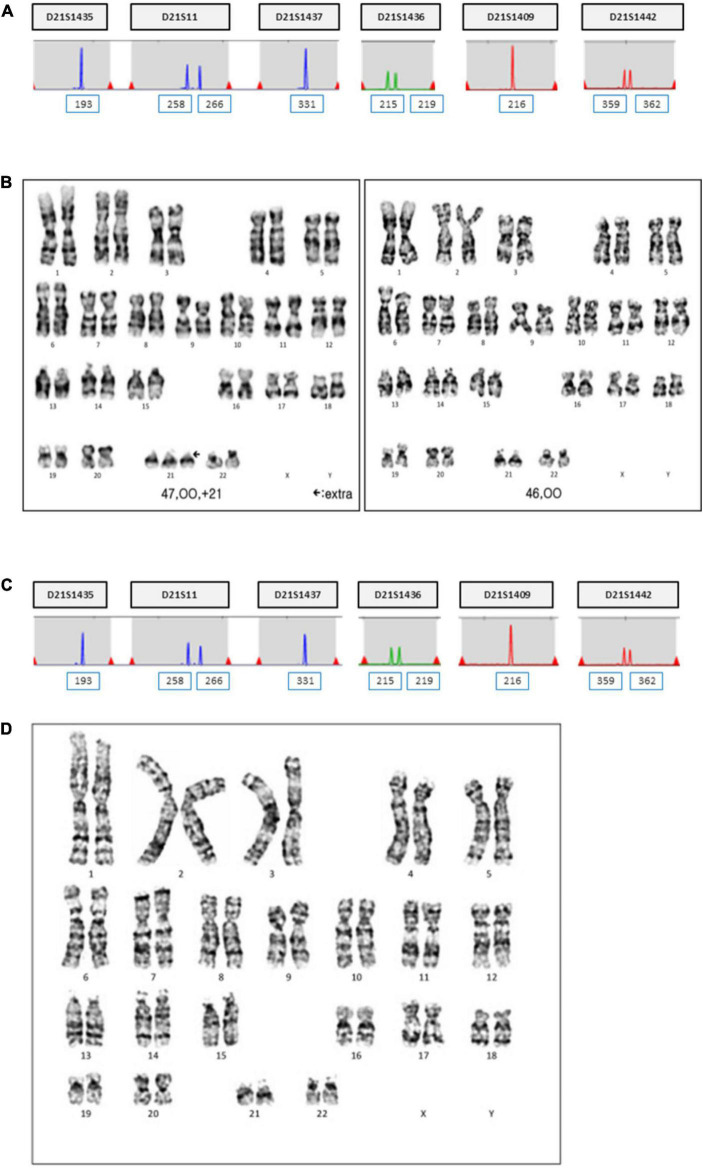
QF-PCR and conventional karyotyping results in case 1. **(A)** QF-PCR analysis for chromosome 21 of uncultured chorionic villi. **(B)** Conventional karyotype analysis of uncultured chorionic villi; 47, XX, +21[11]/46, XX [39]. **(C)** QF-PCR result for chromosome 21 of uncultured amniocytes. **(D)** Conventional karyotype analysis of cultured amniocytes; 46, XX.

The patient was 30 years old and had a history of four pregnancies including this pregnancy and three spontaneous abortions: 47, XY, +21 in the first pregnancy, 45, X in the second pregnancy, and conjoined twin in the third pregnancy. She and her partner requested prenatal fetal screening for aneuploidy. After adequate genetic counseling, NIPT was performed at 11 + 0 weeks of pregnancy, and the result showed a high risk of trisomy 21 (FF = 6.98%, 21 chromosome Z-score = 3.6) (Figure 1). The patient underwent QF-PCR and karyotyping at 14 + 0 weeks of pregnancy through CVS and exhibited a normal pattern in QF-PCR (Figure 2A) and mosaicism of 47, XX, +21[11] and 46, XX [39] (Figure 2B). Owing to the discrepancy between the NIPT and CVS results, the patient underwent amniocentesis at 15 + 6 weeks, and showed a normal pattern in QF-PCR (Figure 2C) and 46, XX karyotyping in long-term culture (Figure 2D).

**FIGURE 3:**
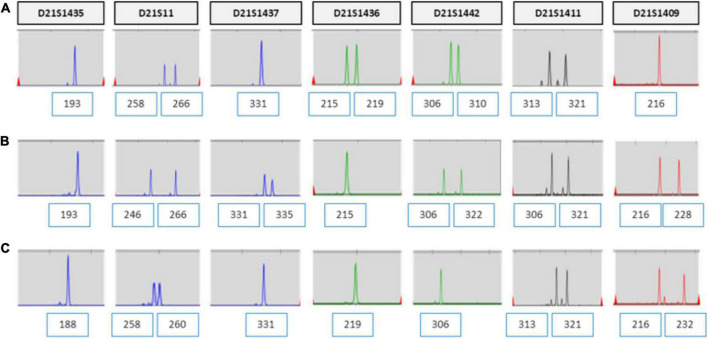
UPD test for chromosome 21 using QF-PCR in case 1. **(A)** Fetus allele in uncultured amniocytes; UPD of chromosome 21 was not detected. **(B)** Maternal allele in peripheral blood. **(C)** Paternal allele in peripheral blood.

**FIGURE 4:**
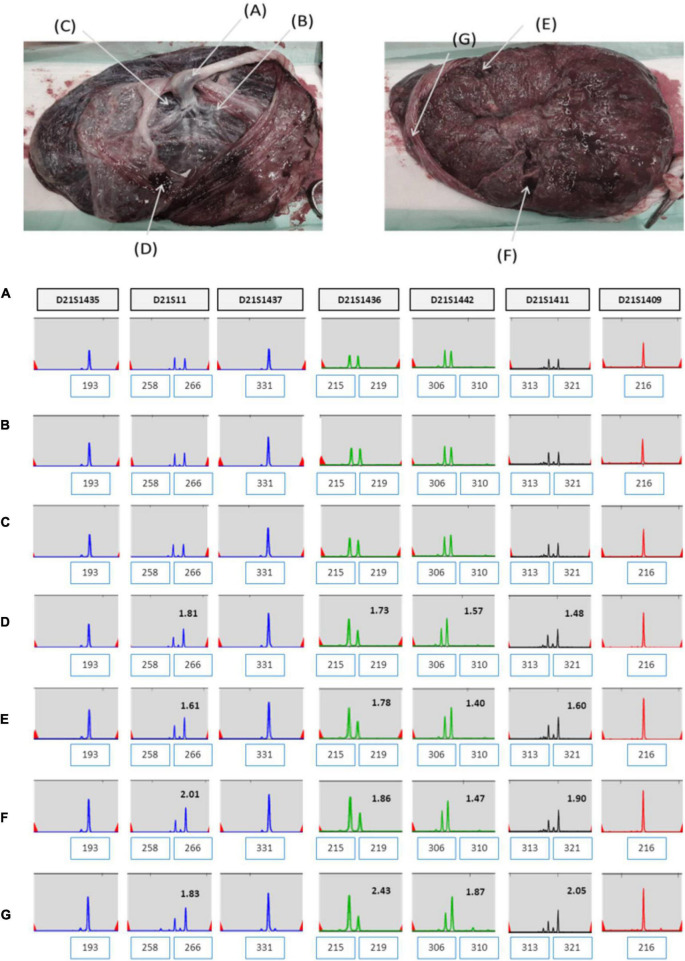
**(A–G)** QF-PCR result for chromosome 21 in each location of placenta in case 1 **(A)** cord blood, **(B)** chorion, **(C)** villus parenchyma (fetal side section), **(D)** villus parenchyma (middle section), **(E)** villus parenchyma (maternal side section I), **(F)** villus parenchyma (maternal side section II), **(G)** amnion.

Short tandem repeat marker tests for chromosome 21 were performed on the parental and amniotic fluid samples to rule out the possibility of UPD. UPD of chromosome 21 was not detected in the amniotic fluid sample (Figure 3). She continued her pregnancy and gave birth to a baby with labor induction at 38 + 5 weeks. After delivery, we sampled 7 × 1 cm^3^ positions of the placenta and checked chromosome 21 using QF-PCR (Figures 4A–G). Chromosome 21 was normal in cord blood and the placenta region close to the fetus. However, trisomy 21 was identified in the placental region close to the mother, including in the amniotic membrane (Figures 4A–G).

### 3.2 Case 2

**FIGURE 5:**
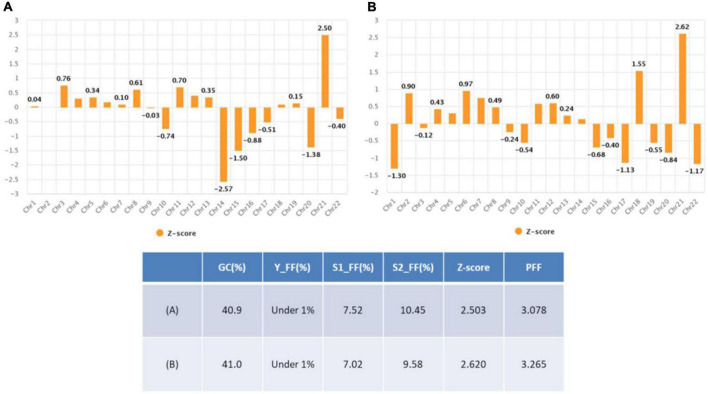
Non- invasive prenatal testing (NIPT) data in case 2 **(A)** 1st trial data **(B)** re-test data.

**FIGURE 6:**
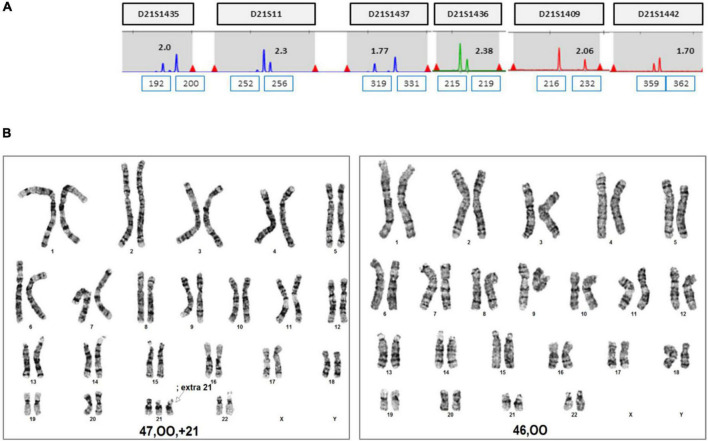
QF-PCR and conventional karyotyping results in case 2. **(A)** QF-PCR result for chromosome 21 of uncultured amniocytes. **(B)** Conventional karyotype analysis of cultured amniocytes; 47, OO, +21[22]/46, OO [4].

**FIGURE 7:**
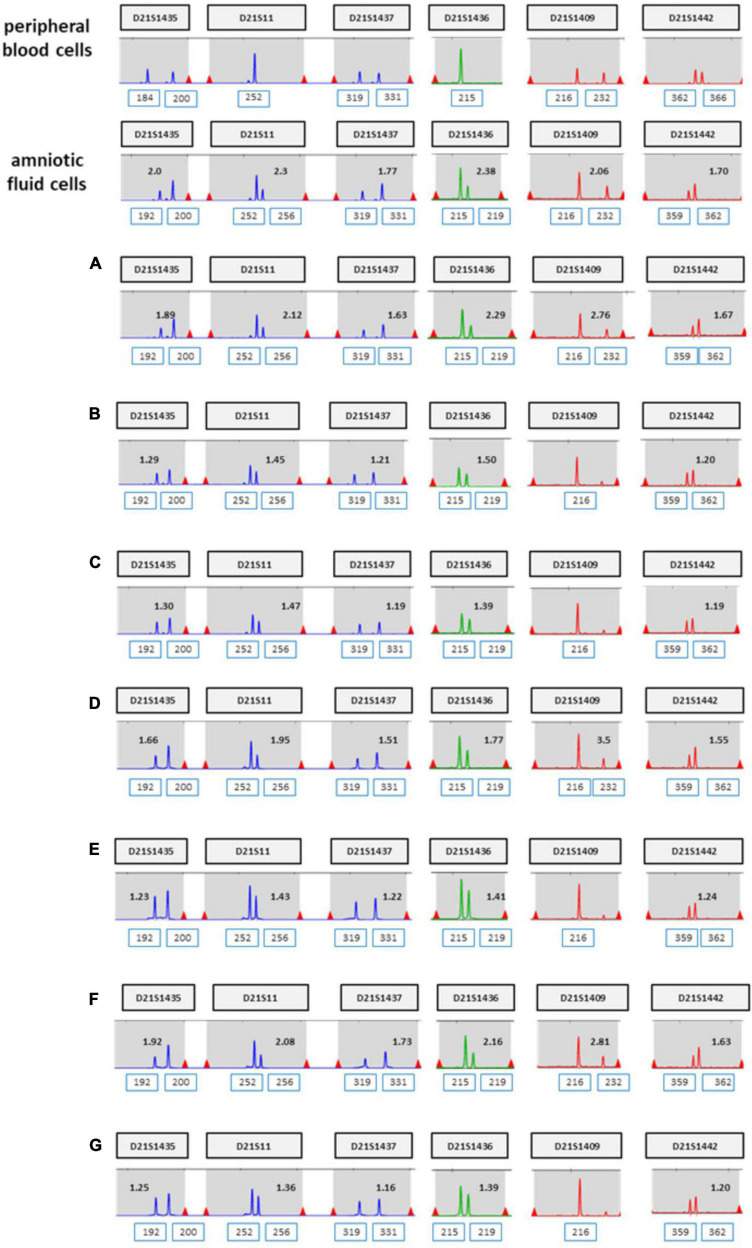
**(A–G)** QF-PCR result for chromosome 21 sampling each location of placenta in case 2 **(A)** cord blood, **(B)** chorion, **(C)** villus parenchyma (fetal side section), **(D)** villus parenchyma (middle section), **(E)** villus parenchyma (maternal side section I), **(F)** villus parenchyma (maternal side section II), **(G)** amnion.

The patient was 37 years old and had a history of five pregnancies, including four spontaneous abortions. She was transferred from another hospital, and the cause of her previous miscarriage could not be confirmed. She underwent NIPT at 12 + 5 weeks of pregnancy, and the results showed no-call data because the FF was relatively low (3.86%). Re-sampling was performed at 16 + 5 weeks of pregnancy, and the results showed a intermediate risk of trisomy 21 (FF = 7.52%, 21 chromosome Z-score = 2.503) (Figure 5A). We repeated the experiment on this sample to exclude a false positive result, and the results again showed a intermediate risk of trisomy 21 again (FF = 7.02%, 21 chromosome Z-score = 2.62) (Figure 5B). We performed an invasive test at 19 weeks of pregnancy through amniotic fluid sampling, followed by QF-PCR and karyotyping. Trisomy 21 was detected by QF-PCR (Figure 6A), and mosaicism of 47, XX, + 21[22]/46, XX [4] was detected by karyotyping (Figure 6B). The patient was counseled with these results and decided to terminate the pregnancy. As the source of cell-free fetal DNA in maternal blood has been shown to be placental in origin, placental biopsies were obtained after abortion. Samples were collected from 7 × 1 cm^3^ positions of the placenta (Figure 7). QF-PCR in one of the samples showed three of the samples showed trisomy 21 (Figures 7A, D, F) and the remaining four samples showed a mosaic pattern (Figures 7B, C, E, G).

### 3.3 Comparison of z-score values for chromosome 21 between two cases and 1,651 data

**FIGURE 8:**
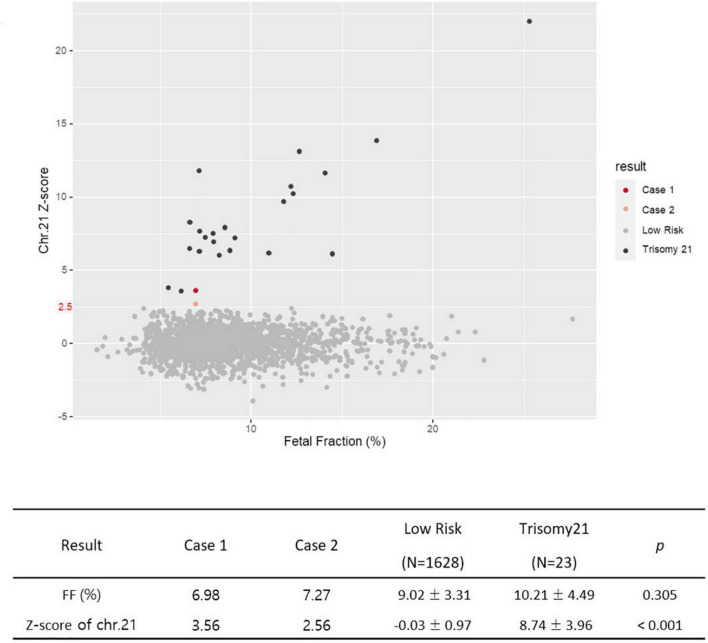
Z-score for chromosome 21 of 1,653 data from Jan 2019 to December 2020.

We summarized the z-scores of chromosome 21 and FF values of total 1,653 including two cases recently acquired in our laboratory. As shown Figure 8, a z-score of 2.5 lies value on the boundary line that separates the low-risk from the intermediate-risk group. The mean FF% of 1,628 low-risk patients was 9.02 ± 3.31, and the z-score value for chromosome 21 was −0.03 ± 0.97. The mean FF% of 23 high-risk group for chromosome 21 was 10.21 ± 4.49 and the z-score was a value of 8.74 ± 3.96. The FF% of the two patients was not significantly different from the other groups, but the z-score showed values that did not belong to the low-risk group and the high-risk group.

## 4 Discussion

Here, we reported two cases of discrepancy between NIPT and invasive tests. In case 1, thorough examinations continued until the patient gave birth, even if trisomy 21 was confined to the placenta. After delivery, the baby weighed 2,760 g, and chromosomal abnormalities, including that in chromosome 21, were not observed. In case 2, the z-score values (2.503 and 2.62) were much lower than FF values (7.52 and 7.02%) in the NIPT results. Hence, it was expected to detect normal or low level of mosaicism pattern, not trisomy 21 in QF-PCR. In contrast, the QF-PCR results of the placental tissue confirmed that the allele pattern differed depending on the location of the placental tissue, and genetic discrepancies existed between the fetus and placenta.

Although there is no way to measure the exact actual chromosomal mosaicism rate, the percentage of mosaic trisomy 21 was evaluated by QF-PCR in the placenta after birth. The rates of trisomy 21 in the placenta of case 1 and 2 was 57.1 and 42.9%, respectively. However, in CVS, it was 22 and 84.6%, respectively. So, our data showed that the level of mosaicism detected by CVS does not always reflect the level present in placenta.

These two patients had a similar experience of several spontaneous abortions, and the results at this pregnancy showed that there was placental mosaicism. Prior reports have suggested that the placental mosaicism influences fetal development and CPM is more likely when placental insufficiency occurs in advanced maternal age (3–5). However, most cases of CPM are undiagnosed and are difficult to identify. Furthermore, since the patient in case 1 was 30 years old, which is a relatively young age, further studies at the molecular genetic level are required to determine the other putative factors apart from age.

Cell-free DNA-based prenatal screening, also known as non-invasive prenatal testing (NIPT), shows excellent sensitivity and specificity for detecting trisomy 21 compared to other screening methods (detection rate is greater than 99%, and the false-positive rate is less than 0.1%) (6). However, at the same time, extremely low but consistent false-negative cases have been reported (7–9). A low trisomic fraction relative to FF may suggest CPM or complete trisomy in fetuses with normal placental cells (10). Mitotic CPM occurs in normal diploid zygotes, and errors after conjugation occur in placental cell lineages, which usually lead to local areas of placental trisomy and low-level mosaicism. In contrast, meiotic CPM occurs in trisomic zygotes, wherein a trisomy rescue event occurs at the beginning of fetal development. In these cases, the fetus is usually diploid, and the placenta is mosaic or completely aneuploid. However, a risk of mosaicism in the fetus depends on the timing of the loss of trisomy in the embryonic cell lineage (5). There may also be a risk of fetal UPD after trisomy rescue, depending on the origin of the missing chromosome.

Fetal cfDNA in maternal peripheral blood originates from trophoblasts and is mainly composed of placental DNA (11–13). NIPT is widely used as an alternative to ultrasonography or invasive fetal testing. However, discrepancies in genetic information between placental and fetal tissues may affect the NIPT outcome, leading to inaccurate results. False-positive NIPT results have been consistently reported and have become concern in recent years (8, 14–16). Additionally, the mosaic condition of the placenta may reduce the measurement accuracy and lead to false-negative results. Therefore, the level of mosaicism is a vital factor in NIPT. Given the influence of the placenta on NIPT, the results should be interpreted in conjunction with various clinical tests based on comprehensive background information.

If the Z-score values of the boundary between low risk and intermediate risk are obtained from the NIPT test results, it is necessary to check whether the Z-score values are consistent even after repeated experiments. If so, a confirmation invasive test is necessary, considering the possibility of mosaicism. We suggest that this approach is optimal for obtaining accurate results and exclude false positives and false negatives. These two cases reaffirm the importance of complementary verification testing following NIPT.

## Data availability statement

The datasets presented in this article are not readily available to protect patient confidentiality and privacy. All data generated or analyzed during this study are included in this article.

## Ethics statement

We obtained written informed consent for participation in the study from 2 patients, and the study was approved by the Institutional Review Board of the CHA Gangnam Medical Center, CHA University, Seoul, Republic of Korea (Approval number: GCI-2022-04-015). The studies for 1,653 data were approved by the Institutional Review Board of the CHA Gangnam Medical Center, CHA University, Seoul, Republic of Korea (approval number: GCI-20-11). Written informed consent was obtained form the 2 patients for the disclosure of data such as publication of articles about the results.

## Author contributions

SS was obtained by funding for this study. SS and DC conceptualized and designed the manuscript. KK and SK drafted the manuscript. SK and SR collected the samples and performed obstetric work-up. JP and HK analyzed the data and interpreted the findings. HJ, MG, SY, and SB performed the experiments. All authors contributed to the article and approved the submitted version.
